# Oxidative Stress Induced in Nurses by Exposure to Preparation and Handling of Antineoplastic Drugs in Mexican Hospitals: A Multicentric Study

**DOI:** 10.1155/2014/858604

**Published:** 2014-02-26

**Authors:** Leobardo Manuel Gómez-Oliván, Gerardo Daniel Miranda-Mendoza, Paula Anel Cabrera-Galeana, Marcela Galar-Martínez, Hariz Islas-Flores, Nely SanJuan-Reyes, Nadia Neri-Cruz, Sandra García-Medina

**Affiliations:** ^1^Laboratorio de Toxicología Ambiental, Facultad de Química, Universidad Autónoma del Estado de México, 50120 Toluca, MEX, Mexico; ^2^Centro Oncológico Estatal del Instituto de Seguridad Social del Estado de México y Municipios, 50180 Toluca, MEX, Mexico; ^3^Laboratorio de Toxicología Acuática, Departamento de Farmacia, Escuela Nacional de Ciencias Biológicas, Instituto Politécnico Nacional, 07700 México, DF, Mexico

## Abstract

The impact of involuntary exposure to antineoplastic drugs (AD) was studied in a group of nurses in diverse hospitals in Mexico. The results were compared with a group of unexposed nurses. Anthropometric characteristics and the biochemical analysis were analyzed in both groups. Also, lipid peroxidation level (LPX), protein carbonyl content (PCC), and activity of the antioxidant enzymes superoxide dismutase (SOD), catalase (CAT), and glutathione peroxidase (GPx) were evaluated in blood of study participants as oxidative stress (OS) biomarkers. The group of occupationally exposed (OE) nurses consisted of 30 individuals ranging in age from 25 to 35 years. The control group included 30 nurses who were not occupationally exposed to the preparation and handling of AD and whose anthropometric and biochemical characteristics were similar to those of the OE group. All biomarkers evaluated were significantly increased (*P* < 0.5) in OE nurses compared to the control group. Results show that the assessment of OS biomarkers is advisable in order to evaluate exposure to AD in nurses.

## 1. Introduction

AD have been reported to induce OS as a mechanism of toxicity. Free radicals formed during this process interaction with macromolecules to induce LPX, as well as oxidation of proteins and of puric and pyrimidine bases of deoxyribonucleic acid (DNA) [1–7]. However, there is a group of antioxidant enzymes such as SOD, CAT, GPx, and glutathione reductase which inhibit oxyradical formation thus aiding in the process of detoxification of these substances in the body [1, 2].

AD should be prepared in a biological safety cabinet designed and operated to ensure protection of the product being handled as well as of nurses and the environment. In all cases, health care workers should receive formal training so that, besides being aware of the risk involved, they can minimize it with appropriate work methods. Exposure of health professionals to this type of pharmaceuticals depends not only on the number of preparations performed each day but also on individual work procedures as well as the precautions taken in handling these agents. The lack of a centralized unit for formal training in the preparation and handling of AD implies a lower level of protection against the potential toxicity of these agents.

Diverse pathologies have been reported in nurses and pharmacy personnel who handle and prepare AD, among others; these pathologies include leukemia, impaired reproductive activity, spontaneous abortion, genotoxicity, cytotoxicity, carcinogenicity, and lymphocyte DNA damage [8–18].

It is important to mention that health professionals in Mexico in charge of preparing and handling AD do not for the most part receive formal training nor are they provided special areas equipped for handling these agents.

Some studies have evaluated OS parameters in nurses. Ulas et al., in 2012, assessed changes in OS parameters, anxiety indexes, and metabolic activities of the nurses in day and night shifts. These parameters were measured in ordinary service and intensive care unit. They found that in ordinary service and intensive care unit nurses, OS parameters, anxiety indexes, and metabolic activities were not different and all nurses suffer the similar effects of the shifts both in day and night. However, there are no reports in the literature indicating the evaluation of OS biomarkers in nurses occupationally exposed to AD preparation and handling [19, 20].

The goal of this study was to evaluate OS by means of LPX, PCC, SOD, CAT, and GPx activities in OE nurses regarding the preparation and handling of AD in different hospitals in Mexico and to determine if OS is a potentially reliable early warning biochemical marker for toxicity assessment in these health care professionals.

## 2. Material and Methods

### 2.1. Selection of Subjects

The transversal and multicentric study was conducted on OE nurses regarding preparation and handling of AD and nurses unexposed to these conditions, who work in different hospitals in the state of Mexico including the Centro Oncológico Estatal ISSEMyM, DIF Children's Hospital, Clinic 220 of the Instituto Mexicano del Seguro Social (IMSS), and ISSEMyM Mother and Child Hospital in the city of Toluca, as well as the IMSS Family Medicine Unit 231 in Metepec.

The research protocol used complies with guidelines of ethical principles in the Declaration of Helsinki (particularly in those aspects involved in noninvasive procedures for human studies) and was approved by the Ethics in Research Committee of the Centro Oncológico Estatal ISSEMyM, the hospital where the project for the present study was submitted for evaluation and to which nurses from the various hospitals and clinics participating in the study were directed.

The initial selection criteria were based on the face-to-face questionnaire. From the started selected group, subsequent inclusion/exclusion criteria were applied (detailed below).

Questionnaire data were collected by two staff members who were trained by the study investigation in participant recruitment, interview content and techniques, the safe handling of the biological samples, and ethical issues related to the study. Each interview was carried out on the day when the blood extractions were performed and required approximately 40 min. The questionnaire includes information on their lifestyle (age, place of residence, birthplace, sleep and rest habits, diet, and physical activities) and employment history (years in an AD preparation-related job and use of protective equipment).

Just before extraction of the sample a complete medical interview was carried out in both selected groups. All the nurses included in this study were free from neoplasias, osteoarticular degenerative diseases, any kind of autoimmune diseases, chronic infections of any etiology (viral, bacterial, or fungal), allergy in any degree, nutritional disorder (such as dislipemias and malnutrition), neurodegenerative diseases, heart diseases under pharmacological treatments, and endocrine illnesses. Excessive smokers (more than 10 cigarettes per day) and alcohol consumers were excluded.

Sampling was nonprobabilistic, opportunistic, sequential, consecutive, and by intact groups. The sample size for each group was 30 individuals, taking into account OE nurses regarding AD preparation and handling and nurses unexposed to these conditions for a total study population of 60 nurses.

Nurses evaluated were invited to participate in the study. They were informed of the characteristics of the study and of the need to take a blood sample from each. Individuals agreeing to take part in the study signed an informed consent letter.

### 2.2. Study Groups

Based on questionnaire responses and inclusion and exclusion criteria, study participants were divided into two groups: OE and unexposed or control.

Nurses in the OE group were selected according to the following criteria: more than two years in an AD preparation-related job and 25 to 35 years of age. Individuals receiving radiation treatment or chemotherapy were excluded from the study.

The control group was formed by nurses who did not come into contact with AD, were similar in socioeconomic characteristics and age to OE participants, and whose work activity did not involve the preparation or handling of AD. These volunteers were initially contacted at the Centro Oncológico Estatal ISSEMyM.

### 2.3. Baseline Definitions and Measurements

Anthropometric measurements were performed according to a standard protocol. Blood pressure (BP) was measured in the morning after 10 min of rest in the sitting position. Abdominal circumference was measured horizontally at the umbilical level at the end of normal expiration. Body mass index (BMI) was calculated by body weight (kg/height (m^2^).

Information on their lifestyle, including age, place of residence, birthplace, sleep and rest habits, diet and physical activities, and employment history, years in an AD preparation-related job, and use of protective equipment was obtained by self-reported questionnaires.

### 2.4. Sample Collection

Morning fasting (8 am) blood samples were collected in both groups on the same day using heparin as an anticoagulant (10 UI/mL) in graduated ice-cold polypropylene test tubes. Plasmas were immediately separated by centrifugation (4000 ×g, 10 min) and stored at −80°C until analyzed. The serum was stored at −80°C. All samples were coded at the time of preparation. The following biomarkers were evaluated: LPX and PCC in order to evaluate oxidized protein content and activity of the antioxidant enzymes SOD, CAT, and GPx.

Other blood samples were collected using EDTA (5.0 mmol/L) as an anticoagulant for use in hemoglobin determination. Hemoglobin level was used to express results of OS markers.

### 2.5. Biochemical Analysis

The activity of aspartate aminotransferase (AST), alanine aminotransferases (ALT), alkaline phosphatase (ALP), and total bilirubin were determined to evaluate hepatic performance. Renal function was evaluated by plasma creatinine and urea concentrations. Also serum glucose and triglycerides were determined. These determinations were performed using commercial kits from Fluka-Sigma-Aldrich, Toluca.

### 2.6. Determination of OS Status

#### 2.6.1. Determination of LPX

LPX was determined using the thiobarbituric acid-reactive substances method (Büege and Aust, 1978) [21]. To 500 *μ*L blood was added Tris-HCl buffer solution with pH 7.4 (Sigma-Aldrich, St. Louis) until a 1-mL volume was attained. Samples were incubated at 37°C for 30 min; 2 mL TBA-TCA reagent (0.375% thiobarbituric acid (Fluka-Sigma-Aldrich, Toluca) in 15% trichloroacetic acid (Sigma-Aldrich, St. Louis)) was added and samples were shaken in a vortex. They were then heated to boiling for 45 min, allowed to cool, and the precipitate removed by centrifugation at 3,000 ×g for 5 min. Absorbance was read at 535 nm against a reaction blank. Malondialdehyde (MDA) content was calculated using the molar extinction coefficient (MEC) of MDA (1.56 × 10^5^ M/cm). Results were expressed as *μ*mol MDA/mg hemoglobin.

#### 2.6.2. Determination of PCC

PCC was determined using the method of Levine et al. [22]. To 100 *μ*L of supernatant was added 150 *μ*L of 10 mM DNPH in 2 M HCl and the resulting solution was incubated at room temperature for 1 h in the dark. Next, 500 *μ*L of 20% trichloroacetic acid was added and the solution was allowed to rest for 15 min at 4°C. The precipitate was centrifuged at 16,000 ×g for 5 min. The bud was washed several times with 1 : 1 ethanol : ethyl acetate, then dissolved in 1 mL of 6 M guanidine solution (pH 2.3) and incubated at 37°C for 30 min. All reagents were obtained from Sigma-Aldrich, St. Louis. Absorbance was read at 366 nm. Results were expressed as *μ*mol reactive carbonyls formed (C=O)/mg hemoglobin, using the MEC of 21,000 M/cm.

#### 2.6.3. Determination of SOD Activity

SOD activity was determined by the Misra and Fridovich (1972) method [23], which is based on inhibition of adrenaline autoxidation at pH 10.2 in erythrocyte lysates free of hemoglobin and other proteins. In a quartz cuvette were placed 150-*μ*L aliquots of homogenate (obtained from 500 *μ*L total blood in 2 mL distilled water, sonicated for 15 min and then supplemented with 2.5 mL of 1 : 1 ethanol : chloroform). Addition was then made of 750 *μ*L of carbonate buffer solution with pH 10.2 (50 mM sodium bicarbonate, 0.1 mM EDTA, adjusted to pH 10.2 with Na_2_CO_3_ in powdered form) and 600 *μ*L adrenaline (30 mM) in 0.05% acetic acid. All reagents were from Sigma-Aldrich, St. Louis. Absorbance was read at 0 s, 30 s, and 5 min, at 480 nm. Absorbance was read at 480 nm after 30 s and 5 min. Results were expressed as UI/mg Hb. Estimates were derived by the formula [SOD] = (*A*_30 s_ − *A*_5min⁡_)∗(*A*_0_/MEC), where the MEC of adrenaline is 21/M/cm.

#### 2.6.4. Determination of CAT Activity

CAT activity was quantified by the Radi et al. method [24], which is based on disappearance of H_2_O_2_ as a result of CAT action through change in absorbance per minute. To 20 *μ*L erythrocyte homogenate plus 1 mL of isolation buffer solution (0.3 M sucrose; 1 mM HEPES; 5 mM KH_2_PO_4_ adjusted to pH 7.4) (Vetec-Sigma-Aldrich, St. Louis) was added 200 *μ*L H_2_O_2_ (20 mM) (Vetec-Sigma-Aldrich, St. Louis), reading absorbance at 0 and 60 s, at 240 nm in quartz cuvettes. Results were expressed as mM H_2_O_2_/mg hemoglobin. Estimates were obtained using the formula [H_2_O_2_] = (*A*_0 s_ − *A*_60 s_)/MEC, where the MEC of H_2_O_2_ is 0.043/mM/cm.

#### 2.6.5. Determination of GPx Activity

GPx activity was determined by the Gunzler and Flohe-Clairborne method [25]. To 100 *μ*L of supernatant was added 10 *μ*L glutathione reductase (2 U glutathione reductase, Sigma-Aldrich) plus 290 *μ*L reaction buffer (50 mM K_2_HPO_4_ (Vetec), 50 mM KH_2_PO_4_ (Vetec) with pH 7.0, 3.5 mM reduced glutathione (Sigma-Aldrich), 1 mM sodium azide (Sigma-Aldrich), and 0.12 mM NADPH (Sigma-Aldrich)) and 100 *μ*L H_2_O_2_ (0.8 mM, Vetec), prior to reading absorbance at 340 nm at 0 and 60 s. Enzyme activity was estimated using the equation GPx concentration = (*A*_0_ − *A*_60_)/MEC, where the MEC of NADPH is 6.2 mM/cm. Results were expressed as mM NADPH/g hemoglobin.

### 2.7. Determination of Hemoglobin

Hemoglobin was determined using a Beckman Coulter AcT Diff hematology analyzer.

### 2.8. Statistical Analysis

This was a transversal study designed to compare analytical data between two samples. Processing and scoring of the samples from exposed and control groups were immediately performed blind and concurrently. At the end of the study, the analytical data and the results obtained from the questionnaire were linked for statistical analyses. All data were expressed as mean ± standard deviation (SD). Student's *t*-test or the *χ* ^2^ test (depending on the type of variable tested) was used for analyzing the results. However, due to the fact that some biochemical parameters may not follow a normal distribution (as judged by Kolmógorov-Smirnov test) the nonparametric Wilcoxon-Mann-Whitney test was also employed (although with equivalent final conclusions). A probability value of *P* < 0.05 was considered to be statistically significant. All analyses were performed using Statistical Package of SPSS version 17.0 for Windows (SPSS, Chicago, IL, USA).

## 3. Results

### 3.1. General Characteristics of the Study Population

The total number of OE nurses was 30; 100% were women, with a mean age of 32 years (range 25–35 years). Control group individuals number was 30; 100% were women, with a mean age of 34 years (range 25–35 years) (Table 1).

Mean time in an AD-related job for OE participants was 4 years (range 2–9 years), suggesting chronic exposure to a wide spectrum of AD including cisplatin, etoposide, gemcitabine, doxorubicin, docetaxel, paclitaxel, vinorelbine, and carboplatin. As regards the use of protective equipment during work, 100% of OE participants said they did not use facemasks, gloves, surgical caps, and protective eyewear or lab coats.

Since none of the nurses in the OE group use protective equipment, they come in greater contact with diverse AD via any one of the potential absorption routes (dermal, inhalatory, digestive, or through the mucosa) which, combined with different temperature gradients and lack of adequate ventilation, poses increased risks to their health.

It is worth noting that in the lifestyle questionnaire, 16 OE group nurses reported working a second shift in private hospitals, where they performed similar activities but with fewer safety measures.

The control group did not carry out any activities associated with AD preparation or handling.

### 3.2. Baseline Definitions and Biochemical Markers

Table 1 shows the main anthropometric characteristics of the study subjects. No significant differences (*P* > 0.05) were observed between OE and unexposed nurses concerning age, BMI, and systolic and diastolic blood pressure.

The biochemical markers, triglycerides, serum glucose, AST, ALT, ALP, total bilirubin, urea, and creatinine also were evaluated. The results in both OE and unexposed groups were within the range of reference values established for the World Health Organization and the kits. No significant differences were observed between OE and unexposed nurses (*P* > 0.05).

### 3.3. Oxidative Stress Markers

In order to assess the exposure degree to AD, the OS markers were measured as typical OS biomarkers.  Table 2 shows the results of LPX obtained in blood samples of the study population. A significant increase (*P* < 0.05) in the OE group (252.6%) compared to the control group was observed in this biomarker. PCC results in the OE group show a significant 218.8% increase compared to the control group (*P* < 0.05). The results of antioxidant status were also significantly altered. A marked increase in SOD activity was found in nurses in the OE group (75.5%) compared to control group individuals (*P* < 0.05). A 166.6% increase in CAT activity occurred in the OE group with respect to the control group (*P* < 0.05) and was statistically significant. Finally, GPx results (Table 2) in the group of OE nurses show a significant 367.7% increase compared to the control group (*P* < 0.05).

## 4. Discussion

Health parameters and OS markers were compared between OE nurses and unexposed or control. The results in OE nurses of anthropometric characteristics, such as age, BMI, and systolic and diastolic blood pressure, as well as the biochemical markers, triglycerides, serum glucose, AST, ALT, ALP, total bilirubin, urea, and creatinine showed not significant differences compared with unexposed group.

Referring to the results of OS status in the present study, they show increases in LPX and PCC in the group of OE nurses regarding the preparation and handing of AD, with respect to the control group (*P* < 0.05). Neoplastic disease studies reveal that treatment with AD increases OS and reduces plasma levels of vitamins C and E as well as of glutathione peroxidase [26].

Diverse AD have been associated with OS. For example, cisplatin induces formation of reactive oxygen species (ROS) in mitochondria, eliciting oxidative alterations in lipids, proteins, and DNA of this organelle [27], while doxorubicin-induced cytotoxicity has been associated with ROS production and in particular to presence of the superoxide anion radical and of hydrogen peroxide [28, 29]. This pharmaceutical is also able to produce reactive nitrogen species (RNS) such as peroxynitrite [30]. The oxidant peroxynitrite is known to induce protein oxidation and nitration in the absence of GSH, eliciting mitochondrial dysfunction and eventually leading to irreversible damage and severe loss of cellular ATP [31]. It is worth noting that both medications are prepared, handled, and administered by nursing personnel in hospitals participating in the present study.

The increases in LPX and PCC found in our study may be explained by an increase in the number of radical species produced by the biotransformation of AD in OE nurses, such as superoxide anion and hydrogen peroxide, which are known to attach to membrane lipids, inducing their lipid peroxidation. Similarly, increased peroxynitrite concentrations may oxidize directly the prosthetic protein group or else react directly with the peptide chain, leading to conformational and functional changes with severe biological consequences for the individual [32].

Paradoxically, oxidative stress induced by oxidative metabolism of antineoplastic drugs interferes with the tumoral growth produced in different types of cancer, since one of the indicators of this process—increased lipid peroxides—favors the prolongation of cell quiescence (G 0 phase). The problem lies in the fact that cytostatic or chemotherapeutic agents act while malignant cells are in constant replication, not when they are quiescent [33–36].

Likewise, antioxidant capability has been reported to be greater in tumoral cells than in normal cells [34], but this effect is surpassed by the OS induced by AD. Short-lived cells or cells with higher renewal rates which are constantly being regenerated are the most affected, in addition to the fact that there are other undesirable effects associated with free radical generation, such as doxorubicin-induced cardiac toxicity (rapid heartbeat, heart failure), bleomycin-induced pulmonary fibrosis, and cisplatin-induced ototoxicity [37–39].

During a person's lifetime, a sophisticated antioxidant network counteracts the deleterious action of ROS on macromolecules [40]. Cells synthesize some of their own antioxidants, as do also SOD, CAT, and GPx as well as peptides with thiol groups, such as glutathione (GSH) and the thioredoxin family. These systems play a major role in the ability of the body to respond to the oxidative challenge of using molecular oxygen to drive reactions that yield the necessary energy.

Increased ROS production is known to be associated with increases in antioxidant enzyme activity. A marked increase in SOD activity occurred in our study in the OE group (75.5%) compared to the control group (*P* < 0.05). Comparison of CAT activity results between study groups found a 166.6% increase of this activity in the OE group, which differed significantly from activity in the control group (*P* < 0.05). Finally, GPx results in the OE group showed a significant 367.6% increase compared to the control group (*P* < 0.05).

SOD is the first mechanism of antioxidant defense and the main enzyme responsible for offsetting toxic effects is induced by the presence of ROS, particularly the superoxide ion, which is formed as a minor product of mitochondrial respiration. Increased SOD activity in our study may be explained by high levels of the superoxide anion radical, which can stimulate this activity. It is well known that the enzyme SOD is known to transform O_2_^∙^ to H_2_O_2_.

Subsequently, the enzyme CAT takes part in the catalytic reaction that decomposes two molecules of the hydrogen peroxide—formed by dismutation of superoxide—into water and oxygen, without the use of cofactors. This function is shared with GPx which uses GSH as a reducing agent [41].

The increase in CAT and GPx may be due to higher levels of hydrogen peroxide, since the oxidative metabolism of AD, such as doxorubicin, to which nurses in our study were exposed, is known to increase the levels of peroxide, which is a specific substrate of GPx.

Similar results of our study were found by Ulas et al. in 2012; they observed that in ordinary service and intensive care unit, the nurses in day and night shifts presented values of total antioxidant status of 0.95–1.01 *μ*mol H_2_O_2_. These values were similar to those found in the activity of catalase in nurses unexposed to AD (1.2 mM H_2_O_2_/mg Hb) [19, 20]. However, OE nurses showed a significant increase from baseline of unexposed nurses to AD.

The increases in HPC, LPX, and PCC in the present study may explain the increases observed in the activity of antioxidant enzymes, as a mechanism of defense against oxidative damage.

Our results showed that OE nurses were more susceptible to oxidative stress than unexposed nurses. No significant differences were found in both groups with respect to biochemical markers evaluated, to explain OS induced in OE nurses. Neither anthropometric characteristic explain OS induced in the exposed group. For these reasons, we believe that OS induced in OE nurses may be explained by exposure to AD.

## 5. Limitations

Certain limitations of the present study should be considered. First, a kinetic used several times must be performed for the different biomarkers of OS to be evaluated. Second, determine AD concentrations in blood of OE nurses and perform a correlation between AD concentration and OS parameters in OE nurses. Third, the sample size was relatively small. Therefore, these results should be verified with large-scale, multicenter prospective cohort studies.

## 6. Conclusions

OE nurses to AD preparation and handling are at potential risk of increasing their levels of OS by not taking preventive and protective measures. Determination of a set of OS biomarkers is important for early detection of their toxic effects in order to prevent health damage in the exposed population.

## Figures and Tables

**Table 1:** Demographical and anthropometric characteristics and the biochemical analysis in control and occupationally exposed groups.

Parameter	Control group	Occupationally exposed group	RV
Age (years)	32 (25–35)	34 (25–35)	
BMI (Kg/m^2^)	21.6 ± 2.1	22.1 ± 2.3	18.5–22.9
Systolic blood pressure (mmHg)	125 ± 12	121.6 ± 10.3	<120
Diastolic blood pressure (mmHg)	77 ± 8	80.5 ± 13.2	<80
Triglycerides (nmol/L)	2.1 ± 0.6	2.6 ± 0.9	<2.82
Serum glucose (nmol/L)	6.1 ± 0.9	5.8 ± 0.7	<7.8
AST (UI/mL)	7.6 ± 0.8	8.3 ± 1.1	<12
ALT (UI/mL)	9.2 ± 0.9	9.8 ± 1.3	<12
ALP (UI/L)	110.3 ± 8.3	122.5 ± 9.6	68–240
Total bilirubin (mg/L)	4.5 ± 0.6	5.1 ± 0.8	<10
Urea (g/L)	0.4 ± 0.06	0.35 ± 0.08	0.20–0.45
Creatinine (mg/L)	11.2 ± 0.9	12.9 ± 1.3	8–14

Data were expressed as the mean ± SD. Results were obtained using commercial kits as detailed in Section *Biochemical analysis*. Reference values (RV) are those established for the World Health Organization and the kits.

BMI: body mass index; AST: aspartate amino transferase; ALT: alanine amino transferase; ALP: alkaline phosphatase.

**Table 2:** Oxidative stress markers in control and occupationally exposed nurses groups.

Biochemical marker	Control group	Occupationally exposed group	*P* value
LPX (*μ*mol MDA/mg Hb)	1.9 ± 0.05	4.8 ± 0.14*	*P* < 0.05
PCC (*μ*mol carbonyls/mg Hb)	1.6 ± 0.07	3.5 ± 0.08*	*P* < 0.05
SOD (UI/mg Hb)	4.5 ± 0.09	7.9 ± 0.07*	*P* < 0.05
CAT (mM H_2_O_2_/mg Hb)	1.2 ± 0.02	2.0 ± 0.05*	*P* < 0.05
GPx (mM NADPH/mg Hb)	5.1 ± 0.06	18.75 ± 0.09*	*P* < 0.05

Data were expressed as the mean ± SD. Values significantly different compared to control group were indicated with *(*P* < 0.05). LPX: lipid peroxidation level; MDA: malondialdehyde; Hb: hemoglobin; PCC: protein carbonyl content; SOD: superoxide dismutase activity; CAT: catalase activity; GPx: glutathione peroxidase activity.
